# Predictive modelling of TBARS changes in the intramuscular lipid fraction of raw ground pork enriched with plant extracts

**DOI:** 10.1007/s13197-021-05187-1

**Published:** 2021-06-29

**Authors:** Anna Maria Kaczmarek, Małgorzata Muzolf-Panek

**Affiliations:** Department of Food Quality and Safety Management, Faculty of Food Science and Nutrition, Poznań University of Life Sciences, 31, 60-624 Poznań, Poland

**Keywords:** Lipid oxidation, Spice extracts, Arrhenius model, Log-logistic model, Neural network

## Abstract

The aim of the study was to develop and compare the predictive models of lipid oxidation in minced raw pork meat enriched with selected plant extracts (allspice, basil, bay leaf, black seed, cardamom, caraway, cloves, garlic, nutmeg, onion, oregano, rosemary and thyme) by investigation TBARS values changes during storage at different temperatures. Meat samples with extract addition were stored under various temperatures (4, 8, 12, 16, and 20°C). TBARS values changes in samples stored at 12°C were used as external validation dataset. Lipid oxidation was evaluated by the TBARS content. Lipid oxidation increased with storage time and temperature. The dependence of lipid oxidation on temperature was adequately modelled by the Arrhenius and log-logistic equation with high R^2^ coefficients (0.98–0.99). Kinetic models and artificial neural networks (ANNs) were used to build the predictive models. The obtained result demonstrates that both kinetic Arrhenius (*R*^2^ = 0.83) and log-logistic (*R*^2^ = 0.84) models as well as ANN (*R*^2^ = 0.99) model can predict TBARS changes in raw ground pork meat during storage.

## Introduction

The consumption of meat in the world is still growing and meat is perceived as one of the most important sources of high-quality protein in the human diet. Pork contains proteins of high biological value, exogenous amino acids, B vitamins, hem iron and other microelements (Bohrer 2017). Lipids are a significant component of all types of meat and are responsible for some desirable properties of meat. They are also very important for the taste and aroma profile of meat, increasing its tenderness and juiciness.

Lipid oxidation is the major process leading to deterioration of meat and meat products by shortening the shelf-life (Min and Ahn 2005). Among the chemical processes, lipid oxidation is a process that significantly reduces the storage stability of meat and meat products. Lipid oxidation is one of the main reasons for reducing the nutritional properties and safety of meat and meat products. Some authors state that one of the most important problems associated with lipid oxidation is the generation of harmful compounds, which are associated with many human pathologies, including atherosclerosis, cancer, inflammation and aging (Angeli et al. 2011; Domínguez et al. 2019; Fan et al. 2019; Sottero et al. 2019; Huang and Ahn 2019).

Lipid oxidation affects colour, structure, nutritional value, taste and aroma leading to rancidity, which is responsible for odours and unacceptable taste, which are important reasons for rejection by consumers. Since both quality and health are the most important factors that influence the consumer's choice of food, the process of lipid oxidation should be minimised, which is very important for the food industry (Amaral et al. 2018).

In meat, lipids undergo oxidation via three main reactions: photo-oxidation, enzymatic oxidation, and autoxidation. Autoxidation of meat lipids is a complex process which results from the high sensitivity of oxidation products to decomposition and reactions with other meat components. Oxidation is also influenced by the presence of catalysts and natural antioxidants in meat, as well as by photo-oxidation occurring simultaneously with auto-oxidation (Domínguez et al. 2019). Changes in lipids with oxygen are the result of free radical reaction, during which the stages of initiation, propagation and termination can be distinguished.

During storage and processing of food products containing fat, especially polyunsaturated acids rich in residues, the oxidation processes taking place in the fat cause adverse organoleptic characteristics and a reduction in the nutritional value of the protein. Fat oxidation products easily interact with proteins, giving resistant to digestive enzymes protein-fat complexes, which leads to a decrease in amino acid assimilability (Viljanen et al. 2004; Hes 2017). Ground meat undergoes disruption in the muscle membranes, which exposes lipid membranes to meat ions and facilitates interactions between unsaturated fatty acids and pro-oxidants. The conditions of raw meat are very important for oxidative changes of meat after cooking because the primary oxidation products or oxidized lipids from the raw meat can continue the oxidation process after cooking (Du et al. 2001). Therefore, preventing lipid oxidation of raw meat is as important as in cooked meat.

One of the methods of preventing lipid oxidation processes in food is the use of antioxidants. The use of synthetic antioxidants for meat preserves is regulated by law and can be used. However, due to consumer distrust of many additives present in food, manufacturers are increasingly replacing them with antioxidants of natural origin (Haugaard et al. 2014; Vallverdú-Queralt et al. 2014; Oswell et al. 2018; Munekata et al. 2020).

Spices have been used in food since ancient times in order to provide it with the desired organoleptic characteristics. The active compounds found in spices have antioxidant properties. Many studies have been carried out concerning the effectiveness and applicability of various types of plant preparations: dried plant parts, water and alcohol extracts and essential oils for meat and products (Hernández-Hernández et al. 2009; Karre et al. 2013; Kaczmarek et al. 2017; Muzolf-Panek et al. 2019; Burri et al. 2020).

Kinetic models as well as models based on artificial neural network are the powerful tool for studying the change in food quality indices during the storage period (Kaczmarek et al. 2015; Stangierski et al. 2019; Limbo et al. 2010; Wang et al. 2020; Wenjiao et al. 2014; Zhang et al. 2020; Guo et al. 2018; Delgado et al. 2016; Panagou et al. 2011; Singh 2009).

Therefore, the aim of the study was to develop and compare the predictive models of lipid oxidation in minced raw pork meat enriched with selected plant extracts by investigation TBARS values changes during storage at different temperatures.

## Material and methods

### Materials

Dried allspice, basil, bay leaf, black seed, cardamom, caraway, cloves, garlic, nutmeg, onion, oregano, rosemary and thyme have been purchased from a local distributor of herbs and spices (Ciecierzyn, Poland). Pork neck (64.7 ± 3.2% moisture, 19.6 ± 0.5% protein, 13.6 ± 2.3% fat) was supplied by a local meat producer (Swarzędz, Poland). Each type of meat was cut, deboned, and minced on site by a 5 mm plate. Then, within half an hour, they were placed in insulated, refrigerated rooms and transported to the laboratory in a chilled state (4–8°C).

### Preparation of spice extracts

Powdered spices (15 g) were mixed with 225 mL of 50% aqueous ethanol in a closed container for 24 h on the magnetic stirrer in the dark. After filtration through 3HW Filtrak filter paper (Filtrak, Niederschlag Bärenstein, Germany) the antioxidant activity and phenolic content of the extract were analysed (Muzolf-Panek et al. 2019). Then, the obtained plant extracts were freeze-dried and used for further studies.

### Antioxidant properties of spice extracts

The radical scavenging activity of the spice extracts was evaluated by the DPPH method according to the procedure described by Sánchez-Moreno et al. (1998) with some modifications. The DPPH• radical scavenging activities of the plant extracts were expressed as Trolox Equivalents Antioxidant Capacity—TEAC (DPPH) values in μM of Trolox equivalent (TE) per g of dry sample. TEAC (DPPH) values were calculated as the ratio of the slope of the linear plot for the scavenging of DPPH• radicals by the extract tested to the slope of the plot for DPPH• radicals scavenging by the antioxidant standard—the water-soluble vitamin E analogue Trolox.

### Content of phenolic compounds in the spice extract

TPC was determined using spectrophotometric method (Singleton and Rossi 1965) with Folin-Ciocalteu reagent and expressed in mg of gallic acid (GAE) equivalents per 1 g of dry matter. Total flavonoid content (TFC) was measured by aluminium chloride method. The amount of 100 μL of spice extract was mixed with 2% aluminium chloride in methanol and left for 15 min. Then the absorbance at 415 nm wavelength was monitored. The results were read from the standard curve for quercetin and expressed in mg quercetin equivalent (QE) per 1 g of extract.

### Meat samples preparation and storage conditions

The frozen dried extract was dissolved in water (60 mL) on the day it was added to the meat (3 kg for each temperature). The concentration of the spice extract expressed in g of powdered spices used for extraction per 100 g of meat was therefore 0.5% (m/m). Fourteen samples were prepared from raw minced pork: one control (C) (meat without extract, only mixed with 60 ml of water) and thirteen samples, namely with allspice, basil, bay leaf, black seed, cardamom, caraway, cloves, garlic, nutmeg, onion, oregano, rosemary, and thyme 0.5% (m/m). Then, each sample was mixed separately for 3 min, placed in a low-density polyethylene bag, and stored at 4, 8 12°C for 13 days and at 16, 20ºC for 5 days.

### Thiobarbituric acid reactive substances (TBARS) determination

TBARS index was used to evaluate the degree of lipid oxidation during storage. The present of TBARS reactive substances is caused by the second stage of auto-oxidation, in which peroxides are oxidised to aldehydes and ketones. TBARS were determined by the method of Mielnik et al. (2006) with some modification (Kaczmarek et al. 2017). The TBARS values were calculated from the standard curve of MDA (malondialdehyde) which was prepared from 1,1,3,3-tetraethoxypropane and expressed in mg of MDA per kg of meat. In order to universalize the obtained models, percentage changes of TBARS values during storage of meat samples at different temperatures were used for their construction.

## Mathematical models

### Kinetic analysis

Analysis of the effects of plant extract addition and storage (time and temperature) on TBARS value changes was performed by fitting experimental values to kinetic models. TBARS data obtained at constant temperature (4, 8, 12, 16 and 20°C) were fitted by a conventional first-order model (Eq. 1)

1 $$TBARS={TBARS}_{0}\mathrm{e}\mathrm{x}\mathrm{p}(kt)$$

where TBARS—value of TBARS index (%), TBARS_0_ is the initial value (100%) at time 0, k is the food quality rate constant (day^−1^) at a given temperature.

Kinetic curves of TBARS reactive substances were drown by plotting the changes in TBARS value (%).

### Temperature dependency

Temperature dependency of the TBARS reactive substance formation in meat lipids could be assessed using Arrhenius equation (Eq. 2):

2 $$k = k_{0} ~exp\left( { - EA} \right)/RT$$

where k (day^−1^) represents the TBARS formation rate, k_0_ is pre-exponential factor, E_a_ (J/mol) is the activation energy, R is the universal gas constant and T is absolute temperature.

The modified logistic Arrhenius equation was given by the equation (Eq. 3):

3 $$lnk={lnk}_{0}-{E}_{a}/RT$$

An alternative for the Arrhenius equation is a log-logistic relationship (Eq. 4).

4 $$k=m'ln(1+exp([c(T-T_{c})]))$$

where c (ºC^−1^), m’ (-), and T_c_ (ºC^−1^) are empirical fit constants and in many cases, it can be assumed that m’ = 1. This equation does not need the concept of activation energy.

### Artificial Neural networks (ANNs)

ANNs used storage conditions (time and temperature) and plant extract addition as input data for the ANN calculation. The datasets were divided into three subsets in a ratio of 2:1:1. These were a training set (a set of samples used to adjust the net- work weights), a validation set (a set of samples used to tune the parameters), and a test set (a set of samples used only to assess the performance to new, unseen observations). The performance of the neural network was confirmed by measuring its performance on a third independent set of data called a test set. The ANN was trained using selected parameters from the data set and was subsequently validated using an independent data set. Multilayer feed-forward connected ANN has been trained with the Broyden-Fletcher-Goldfarb-Shanno learning algorithm (200 epoch). The search for appropriate ANN model was performed using multilayer perceptron (MLP) and radial basis function (RBF) networks. In total, 20 networks were evaluated and the best five were retained. The network structure developed for honey data included an input layer, one hidden layer and an output layer. The input layer made up of 16 neurons, 3–7 neurons in a hidden layer and one neuron in the output layer predicted values of TBARS (%). The sums of squares and the cross-entropy error function were used during the network training process. The success of the model to predict TBARS values was assessed as: training performance as a percentage of the samples in the learning set correctly predicted during the networks learning step; test performance as a percentage of the samples in the testing set correctly predicted during the networks testing step; and validation performance as a percentage of the samples in the validation set (samples not used in the learning and testing steps) correctly predicted by the models during the networks validation step.

### Validation and evaluation of kinetic and ANN models

The external validation was performed. TBARS values changes models at 4, 8, 16 and 20°C were established by combining kinetic analysis and Arrhenius equation or kinetic analysis and log-logistic equation as well as ANN models. TBA reactive substance changes at 12°C were adopted to evaluate the performance of obtained predictive models.

### Regression modelling

To assess the influence of time, temperature, and addition of plant extracts on TBARS formation in meat lipids fraction, multiple linear regression (MLR) was performed. TBA reactive substances increase exponentially therefore a logarithmic transformation was used to linearize this relationship. The general model of Multiple Linear Regression has a following equation (Eq. 5):

5 $$y = \beta \_0 + \beta \_1~x\_1 + \beta \_2~x\_2 \ldots \beta \_k~x\_k + \varepsilon$$

where: y—variable value; β_0_—intercept; β_1 – k_—regression coefficient; x_1-k_—predictors; ε—standard estimation error. To compare the rates (slope of regression equation) of TBARS formation in meat samples with different plant extracts, a multiple linear regression analysis was performed. The comparisons between the coefficients were performed introducing 13 (k-1) dummy variables as predictors to regression analysis. The control samples have not been coded because this is the category with which all other categories will be compared. The significant differences between the regression coefficients were based on the result of the t-test (*P* ≤ 0.05) for dummy variables. Differences were considered significant at the p ≤ 0.05 level.

### Statistical analysis

All analysis was run in triplicate and the results are expressed as mean ± standard deviations (SD). All statistical tests were performed using Statistica 13.3 software (StatSoft, Tulsa, Oklahoma, USA). A significance level of *α* = 0.05 was used.

Values of kinetic parameters were evaluated using non-linear estimation analysis by least-squares criterion with Levenberg–Marquardt algorithm. The goodness of fits of the models was verified based on the determination coefficient (R^2)^ and root-mean-square error (RMSE).

## Results and discussion

### Antioxidant activity and phenolic content of spice extracts

Antioxidant activity of spice extracts is shown in Table 1. The results of antioxidant activity and phenolic content in allspice, bay leaf, black seed, caraway, cardamom, clove and nutmeg were previously published (Muzolf-Panek et al. 2019). The highest content of phenolic compounds and the highest antioxidant activity was recorded for clove extract: 167 mg GAE/g and 1443 µM TE/g respectively. Similar TPC values for clove were obtained by (Assefa et al. 2018; Elhussein et al. 2018). The antioxidant aqueous ethanol (80%) extract of clove exhibit similar but higher phenolic content equal to 230 mg GAE/g (El-Maati et al. 2016). Moreover, allspice, thyme, bay leaf, oregano and basil showed both high antioxidant activity and high phenolic content. The values of TPC were positively correlated with the DPPH• radical scavenging capacity (r = 0.98, *p* = 0) which was noted previously (Muzolf-Panek et al. 2019, 2020).

**Table 1:** Antioxidant activity and phenolic compound content of ethanol in water (1/1v/v/) extracts

Extract	Antioxidant activity	Bioactive compounds content
	TEAC (DPPH) µM TE/g	TPC mg GAE g/DW
Allspice	555 ± 24^g^	31.61 ± 0.81^e^
Basil	134.7 ± 2.3^c^	14.81 ± 0.35^bc^
Bay leaf	231.9 ± 1.5^e^	22.56 ± 0.16^ cd^
Black seed	7.59 ± 0.84^a^	2.46 ± 0.61^a^
Cardamom	5.45 ± 0.35^a^	1.24 ± 0.01^a^
Caraway	20.2 ± 0.6^a^	2.39 ± 0.14^a^
Clove	1443 ± 1^h^	167.2 ± 9.3^f^
Garlic	14.8 ± 1.6^a^	3.6 ± 0,05^a^
Nutmeg	22.22 ± 0.15^ab^	3.89 ± 0.14^a^
Onion	5.74 ± 0.28^a^	7.05 ± 0.58^ab^
Oregano	171.6 ± 5.8^d^	20.7 ± 0.1^ cd^
Rosemary	50.4 ± 3.6^b^	4.66 ± 0.36^a^
Thyme	278.3 ± 16.2	23.5 ± 0.6^d^

All values are mean ± SD of the three replicates

TPC–total polyphenol content,

^(a^^−^^f)^ means with the same superscript within the same column are not different (*p* > 0.05)

### Development of mathematical models for TBA reactive substance formation in ground pork meat

All meat samples were kept under controlled conditions and taken for analysis in appropriate time intervals to allow efficient kinetic analysis of secondary lipids oxidation products measured using TBARS index. The highest regression coefficients values were obtained for logarithmic TBARS value vs. time. Therefore, the first-order reaction model was applied (Eq. 1). Also in rabbit meat during refrigerated and frozen storage, changes in the TBARS index followed a first-order kinetic model (Wang et al. 2020). The effect of temperature was included to the mathematical using Arrhenius (Eq. 3) and Log-logistic (Eq. 4) equations. The predictive models were obtained by integrating Eqs. (1) and (3) and Eqs. (1) and (4).

### Arrhenius models

With the first reaction order and corresponding rate constant derived from chemical kinetics, parameters in Arrhenius models (Eq. 3) were calculated by linear regression (lnk vs. 1/T). The activation energy Ea can be seen as the energy barrier that molecules need to cross in order to be able to react. The proportion of molecules able to do that increases with temperature, which qualitatively explains the effect of temperature on rates. Since the TBARS index was monitored in meat system the concept of Ea as the minimum energy required for the reaction should be discussed very carefully which was mentioned by (Boekel 2009). Activation energy values indicated how sensitive to temperature the samples were. The results are presented in Table 2. The temperature dependency is better described by Log-logistic model than Arrhenius ones with higher average values of determination coefficient equal 0.988 and 0.992, respectively. The highest R^2^ value was noted for Arrhenius parameters obtained based on TBARS changes in meat sample with cardamom extract addition (*R*^2^ = 0.995). Whereas the lowest in meat samples enriched with garlic extract (*R*^2^ = 0.974). For the control sample, Ea was 64.55 kJ/mol and almost the same Ea value (64.7 kJ/mol) was obtained for raw pork tenderloin stored at 4, 15 and 25°C (Meng et al. 2018). E_a_ values for TBARS formation vary from 51.65 kJ/mol for clove-treated samples to 65 kJ/mol for garlic-treated samples. Therefore, the samples can be ordered from the least sensitive to temperature to the most sensitive in the following order: clove < cardamom < caraway < thyme < black seed < nutmeg < oregano < allspice < bay leaf < basil < onion < rosemary < control < garlic. In contrast, using the same plant extracts in a matrix of raw ground beef, the following relationships were obtained: black seed > clove > allspice > oregano > rosemary ≥ basil > bay leaf > onion > cardamom ≈ thyme > garlic > nutmeg ≥ control > caraway (Kaczmarek and Muzolf-Panek 2021a). It can be observed that meat samples with the addition of clove in both cases are among the most sensitive to temperature while meats enriched with garlic extract and control samples are among the least sensitive to temperature.

**Table 2:** Parameters of Arrhenius model and log-logistic model for TBARS values predicting of ground pork with plant extracts during storage at different temperatures

Extracts	Temperature (K)	k	Arrhenius model			Log-logistic model		
			*R*^2^	E_a_	k_0_	*R*^2^	c	T_c_
Allspice	277	0.07334 ± 0.00081	0.9924 ± 0.0006	58,834 ± 339	8.9 × 10^9^ ± 1.2 × 10^9^	0.9966 ± 0.0005	0.0997 ± 0.00036	294.14 ± 17.23
	281	0.09552 ± 0.00118						
	289	0.19964 ± 0.00064						
	293	0.29104 ± 0.00214						
Rosemary	277	0.08209 ± 0.00196	0.983 ± 0.0039	61,333 ± 883	2.9 × 10^10^ ± 1.08 × 10^10^	0.9907 ± 0.0013	0.1094 ± 0.0031	301.34 ± 0.62
	281	0.10839 ± 0.00013						
	289	0.22926 ± 0.00704						
	293	0.34830 ± 0.01267						
Bay leaf	277	0.08662 ± 0.00090	0.9786 ± 0.0018	59,207 ± 412	1.1 × 10^10^ ± 1.910^9^	0.9907 ± 0.0007	0.1039 ± 0.0002	302.02 ± 0.05
	281	0.10659 ± 0.00062						
	289	0.23613 ± 0.00219						
	293	0.33710 ± 0.00131						
Black seed	277	0.07215 ± 0.00012	0.9921 ± 0.0002	56,185 ± 1848	3.3 × 10^9^ ± 2.8 × 10^9^	0.9953 ± 0.0008	0.0946 ± 0.0036	305.72 ± 0.973
	281	0.09225 ± 0,00,116						
	289	0.18412 ± 0.00861						
	293	0.27031 ± 0.01061						
Cardamom	277	0.06906 ± 0.00021	0.9949 ± 0.0006	52,239 ± 108	4.7 × 10^8^ ± 2.1 × 10^7^	0.9957 ± 0.0010	0.0868 ± 0.0002	308.44 ± 0.04
	281	0.08997 ± 0.00011						
	289	0.16608 ± 0.00163						
	293	0.23905 ± 0.00039						
Caraway	277	0.07046 ± 0.00092	0.9935 ± 0.0021	52,453 ± 217	5.2 × 10^8^ ± 4.6 × 10^7^	0.9957 ± 0.00104	0.0876 ± 0.0009	308.06 ± 0.27
	281	0.09024 ± 0.00024						
	289	0.16957 ± 0.00069						
	293	0.24334 ± 0.00239						
Clove	277	0.06834 ± 0,00,007	0.9891 ± 0.0099	51,655 ± 1042	3.8 × 10^8^ ± 1.5 × 10^8^	0.9867 ± 0.0145	0.0860 ± 0.0003	308.86 ± 0.22
	281	0.08858 ± 0.00006						
	289	0.15796 ± 0.00925						
	293	0.23645 ± 0.00026						
Control	277	0.12067 ± 0.0004	0.9917 ± 0.0005	64,549 ± 505	1.7 × 10^11^ ± 3.8 × 10^10^	0.996 ± 0.0012	0.1164 ± 0.0014	296.02 ± 0.16
	281	0.1651 ± 0.001						
	289	0.37407 ± 0.001						
	293	0.54319 ± 0.00722						
Garlic	277	0.08689 ± 0.00032	0.9738 ± 0.0027	65,007 ± 314	1.4 × 10^11^ ± 1.9 × 10^10^	0.9903 ± 0.0032	0.1192 ± 0.0003	299.420 ± 0.13
	281	0.11049 ± 0.00192						
	289	0.25619 ± 0.00264						
	293	0.39282 ± 0.00453						
Nutmeg	277	0.07571 ± 0.00134	0.9897 ± 0.0013	58,393 ± 684	7.4 × 10^9^2.1 × 10^9^ ±	0.9936 ± 0.0016	0.1011 ± 0.0003	303.71 ± 0.03
	281	0.09882 ± 0.00214						
	289	0.20005 ± 0.00019						
	293	0.30057 ± 0.00257						
Onion	277	0.10824 ± 0.00209	0.9899 ± 0.0003	59,926 ± 372	2.2 × 10^10^ ± 3.3 × 10^9^	0.9864 ± 0.0002	0.0965 ± 0.0006	298.94 ± 0.19
	281	0.16924 ± 0.0013						
	289	0.3528 ± 0.00403						
	293	0.44135 ± 0.00562						
Thyme	277	0.07207 ± 0.00022	0.9924 ± 0.0013	52,572 ± 556	5.7 × 10^8^ ± 1.1 × 10^8^	0.9943 ± 0.0025	0.0869 ± 0.0004	307.89 ± 0.13
	281	0.09067 ± 0.00007						
	289	0.17193 ± 0.00506						
	293	0.24861 ± 0.00159						
Basil	277	0.07907 ± 0.00038	0.9862 ± 0.0007	59,854 ± 286	1.4 × 10^10^ ± 1.9 × 10^9^	0.9909 ± 0.0004	0.1055 ± 0.0006	302.28 ± 0.17
	281	0.1081 ± 0.00068						
	289	0.21726 ± 0.00287						
	293	0.32914 ± 0.00318						
Oregano	277	0.07801 ± 0.00056	0.9885 ± 0.0003	58,800 ± 177	8.9 × 10^9^ ± 7.4 × 10^8^	0.9934 ± 0.0005	0.1020 ± 0.0002	303.11 ± 0.06
	281	0.10292 ± 0.00138						
	289	0.21090 ± 0.00363						
	293	0.31159 ± 0.00298						

All values are mean ± SD of the three replicates

The physical meaning of k_0_ is that it represents the rate constant at which all molecules have sufficient energy to react (Ea = 0). The highest k_0_ values were noted for control (k_0_ = 1.7 × 10^11^), while the lowest k_0_ value was 4.7 × 10^8^ for cardamom-treated meat samples.

The Arrhenius model of TBA reactive substances changes in ground pork meat with various plats extracts addition was given in Eq. (6):

6 $$TBARS={TBARS}_{0}*\mathrm{e}\mathrm{x}\mathrm{p}(k*\mathrm{e}\mathrm{x}\mathrm{p}({E}_{a}/RT)*t)$$

where, TBARS—value of TBARS index (%), TBARS_0_ is the initial value (100%) at time 0, k represents the TBARS formation rate, E_a_ is the activation energy, R is the universal gas constant, T is absolute temperature and t is the storage time. The values of k and activation energy (E_a_) are given in Table 2. The goodness of fit of Arrhenius models are presented in Table 3. The average value of adjusted R^2^ between observed and predicted TBARS values was equal 0.917. The highest value of determination coefficient was noted for control (R^2^ = 0.992) whereas the lowest for clove-treated samples (R^2^ = 0,879). Also, the sum of R^2^ (3.97) was higher for control samples in tested temperatures than for the other samples in tested temperatures (Tab. 3).

**Table 3:** The goodness of fit of Arrhenius and Log-logistic models of TBA reactive substances changes in ground pork meat with various plats extracts addition during storage at different temperatures

Extract	Temperature (°K)	Model					
		Arrhenius			Log-logistic		
		R^2^	RMSE	ΣR^2^	R^2^	RMSE	ΣR^2^
Allspice	277	0.9807 ± 0.0040	7.65 ± 0.89	3.682	0.9656 ± 0.0086	10.20 ± 1.43	3.700
	281	0.8287 ± 0.0065	31.73 ± 0.10		0.8728 ± 0.0074	27.34 ± 0.35	
	289	0.9400 ± 0.0023	14.84 ± 0.26		0.9359 ± 0.0022	15.35 ± 0.24	
	293	0.9325 ± 0.0055	21.32 ± 0.76		0.9254 ± 0.0053	22.42 ± 0.67	
Rosemary	277	0.9455 ± 0.0104	16.06 ± 2.10	3.648	0.8821 ± 0.0272	23.60 ± 3.63	3.628
	281	0.8554 ± 0.0089	37.27 ± 1.29		0.8951 ± 0.0026	31.75 ± 0.31	
	289	0.9398 ± 0.0067	18.19 ± 1.70		0.9337 ± 0.0073	19.10 ± 1.76	
	293	0.9068 ± 0.0113	36.36 ± 4.99		0.9175 ± 0.0082	34.20 ± 4.31	
Bay leaf	277	0.9194 ± 0.0089	20.84 ± 1.43	3.627	0.8637 ± 0.0171	27.09 ± 2.06	3.641
	281	0.8332 ± 0.0142	38.06 ± 1.54		0.8993 ± 0.0070	29.57 ± 0.91	
	289	0.9444 ± 0.0012	17.97 ± 0.25		0.9415 ± 0.0006	18.43 ± 0.13	
	293	0.9304 ± 0.0054	29.07 ± 1.51		0.9368 ± 0.0028	27.72 ± 0.91	
Black seed	277	0.9787 ± 0.0008	7.68 ± 0.04	3.679	0.9718 ± 0.0030	8.83 ± 0.61	3.709
	281	0.8245 ± 0.0054	29.95 ± 0.36		0.8705 ± 0.0053	25.73 ± 0.21	
	289	0.9417 ± 0.0028	13.50 ± 0.42		0.9372 ± 0.0027	14.01 ± 0.40	
	293	0.9343 ± 0.0159	18.75 ± 3.81		0.9292 ± 0.0158	19.47 ± 3.76	
Cardamon	277	0.9534 ± 0.0009	10.06 ± 0.10	3.633	0.9659 ± 0.0004	8.61 ± 0.14	3.663
	281	0.8093 ± 0.0054	29.26 ± 0.35		0.8465 ± 0.0053	26.25 ± 0.44	
	289	0.9040 ± 0.0066	15.03 ± 0.42		0.8995 ± 0.0070	15.38 ± 0.43	
	293	0.9130 ± 0.0031	17.39 ± 0.29		0.9003 ± 0.0041	18.62 ± 0.36	
Caraway	277	0.9656 ± 0.0004	8.95 ± 0.17	3.580	0.9710 ± 0.0033	8.21 ± 0.60	3.612
	281	0.8167 ± 0.0020	28.96 ± 0.16		0.8574 ± 0.0046	25.54 ± 0.41	
	289	0.9177 ± 0.0027	14.41 ± 0.14		0.9131 ± 0.0021	14.81 ± 0.06	
	293	0.9332 ± 0.0025	15.59 ± 0.45		0.9218 ± 0.0034	16.86 ± 0.45	
Clove	277	0.9458 ± 0.0034	10.46 ± 0.25	3.518	0.9628 ± 0.0032	8.66 ± 0.33	3.568
	281	0.7810 ± 0.0160	30.08 ± 1.07		0.8299 ± 0.0212	26.49 ± 1.65	
	289	0.8900 ± 0.0243	15.01 ± 2.37		0.8869 ± 0.0209	15.23 ± 2.14	
	293	0.9009 ± 0.0166	17.90 ± 1.48		0.8880 ± 0.0174	19.04 ± 1.44	
Control	277	0.9916 ± 0.0008	15.24 ± 0.55	3.971	0.9782 ± 0.0023	24.55 ± 1.55	3.934
	281	0.9968 ± 0.0008	12.62 ± 1.49		0.9897 ± 0.0046	22.28 ± 5.10	
	289	0.9926 ± 0.0007	14.28 ± 0.92		0.9878 ± 0.0014	18.31 ± 1.37	
	293	0.9905 ± 0.0040	24.56 ± 4.92		0.9784 ± 0.0049	37.58 ± 3.10	
Garlic	277	0.9279 ± 0.0079	20.66 ± 0.97	3.556	0.8220 ± 0.0165	32.49 ± 1.25	3.546
	281	0.8557 ± 0.0133	37.79 ± 0.73		0.9299 ± 0.0013	26.40 ± 1.13	
	289	0.9230 ± 0.0020	23.84 ± 0.59		0.9172 ± 0.0022	24.72 ± 0.60	
	293	0.8496 ± 0.0041	63.09 ± 2.80		0.8774 ± 0.0041	56.97 ± 2.71	
Nutmeg	277	0.9707 ± 0.0064	9.94 ± 1.31	3.683	0.9403 ± 0.0104	14.19 ± 1.57	3.689
	281	0.8392 ± 0.0026	32.86 ± 1.60		0.8850 ± 0.0047	27.80 ± 1.80	
	289	0.9429 ± 0.0007	14.72 ± 0.24		0.9370 ± 0.0002	15.47 ± 0.17	
	293	0.9300 ± 0.0029	23.08 ± 0.79		0.9272 ± 0.0023	23.53 ± 0.53	
Onion	277	0.9562 ± 0.0008	27.90 ± 0.83	3.783	0.9338 ± 0.0064	34.25 ± 0.92	3.761
	281	0.9615 ± 0.0027	40.50 ± 1.92		0.9394 ± 0.0029	50.77 ± 1.27	
	289	0.9515 ± 0.0041	32.25 ± 1.09		0.9515 ± 0.0040	32.27 ± 1.05	
	293	0.9137 ± 0.0052	52.94 ± 1.17		0.9364 ± 0.0011	45.48 ± 1.83	
Thyme	277	0.9701 ± 0.0025	8.81 ± 0.25	3.664	0.9735 ± 0.0022	8.29 ± 0.25	3.695
	281	0.8069 ± 0.0055	30.29 ± 0.55		0.8437 ± 0.0138	27.23 ± 1.29	
	289	0.9368 ± 0.0057	13.21 ± 0.32		0.9333 ± 0.0070	13.57 ± 0.44	
	293	0.9501 ± 0.0016	14.06 ± 0.14		0.9449 ± 0.0014	14.78 ± 0.09	
Basil	277	0.9604 ± 0.0009	12.88 ± 0.25	3.682	0.9192 ± 0.0031	18.40 ± 0.50	3.665
	281	0.8633 ± 0.0077	35.98 ± 0.83		0.8865 ± 0.0072	32.79 ± 0.86	
	289	0.9434 ± 0.0037	16.49 ± 0.40		0.9376 ± 0.0040	17.31 ± 0.40	
	293	0.9149 ± 0.0037	30.79 ± 0.78		0.9217 ± 0.0034	29.53 ± 0.79	
Oregano	277	0.9648 ± 0.0021	11.62 ± 0.52	3.686	0.9352 ± 0.0010	15.76 ± 0.39	3.687
	281	0.8420 ± 0.0080	35.24 ± 0.26		0.8801 ± 0.0065	30.70 ± 0.24	
	289	0.9441 ± 0.0012	15.61 ± 0.38		0.9379 ± 0.0036	16.44 ± 0.37	
	293	0.9350 ± 0.0017	23.80 ± 0.82		0.9335 ± 0.0028	24.06 ± 0.59	

All values are mean ± SD of the three replicates

### Log-logistic model

An alternative to Arrhenius model is the log-logistic model (Eq. 4). Parameters of obtained models are showed in Table 2. The high regression coefficients (R^2^ > 0.98) in all groups indicated that the log-logistic temperature dependency well described this relation in tested samples. Similar fit (*R*^2^ > 0.9) received Bao et al. (2013) who modelled quality changes in Songpu mirror carp (*Cyprinus carpio*) fillets stored at chilled temperatures using Log-logistic model. The highest *R*^2^ value was observed for log-logistic model parameters obtained based on TBARS changes in meat sample with allspice extract addition (*R*^2^ = 0.996). While the lowest in meat samples enriched with onion extract (*R*^2^ = 0.986).

The log-logistic model of TBA reactive substances changes in ground pork meat with various plats extracts addition was given in Eq. (7):

7 $$TBARS={TBARS}_{0}*\mathrm{e}\mathrm{x}\mathrm{p}(\mathrm{ln}\left(1+\mathrm{exp}\left(c*\left(T-{T}_{c}\right)\right)\right)*t)$$

where, TBARS—value of TBARS index (%), TBARS_0_ is the initial value (100%) at time 0, c (ºC-1), and Tc (ºC^−1^) are empirical fit constants and t is the storage time. The values of constants are presented in Table 2. The goodness of fit of log-logistic models are presented in Table 3. The average value of adjusted R^2^ coefficient for observed and predicted TBARS values was equal 0.919. The highest value of determination coefficient was noted for control (*R*^2^ = 0.984) whereas the lowest for clove-treated samples (*R*^*2*^ = 0.886). Also, the sum of R^2^ (3.93) was higher for control samples in tested temperatures than for the other samples in tested temperatures (Tab. 3). The log-logistic models showed a slightly better goodness of fit than Arrhenius models with average sum of R^2^ values equal 3.68 and 3.67, respectively. The obtained results demonstrated that kinetic models could accurately predict changes in the TBARS index in raw minced pork meat samples enriched with plant extracts under various time–temperature conditions. Kinetic models have also been successfully used to predict protein oxidation (expressed by changes in thiol groups) of chicken meat (Kaczmarek and Muzolf-Panek 2021) as well as beef (Muzolf-Panek and Kaczmarek 2021).

## Artificial Neural networks (ANNs)

The best five ANN-MLP networks are presented in Table 4. In neural network obtained for TBARS values the Tanh and logistic functions were used in the hidden layer, while exponential and logistic functions were used in the output layer. The number of neurons in hidden layer vary from 5 to 9. The goodness of fit of all selected networks was very high. The best network was MLP 16–8-1 with the highest adjusted determination coefficient (*R*^2^ = 0.9955) and the lowest RMSE (10.13) values.

**Table 4:** ANN model parameters for TBARS changes in ground pork meat enriched with plant extracts stored at different temperatures

Net parameters	Net structure				
	MLP 16–7-1	MLP 16–9-1	MLP 16–5-1	MLP 16–5-1	MLP 16–8-1
Training accuracy	0.996	0.995	0.995	0.995	0.993
Test accuracy	0.996	0.996	0.993	0.993	0.990
Validation accuracy	0.994	0.993	0.992	0.990	0.988
Training error	89.1	113.5	126.4	133.0	183.4
Test error	88.3	81.3	140.5	142.5	207.0
Validation error	91.1	117.1	136.0	161.4	200.6
Training algorithm	BFGS 146	BFGS 95	BFGS 98	BFGS 96	BFGS 69
Error function	SOS	SOS	SOS	SOS	SOS
Hidden activation	Logistic	Tanh	Tanh	Tanh	Logistic
Output activation	Exponential	Exponential	Logistic	Exponential	Logistic
R^2^	0.9922	0.9873	0.9910	0.9947	0.9955
RMSE	13.36	16.98	14.31	11.00	10.13

### Validation and evaluation of quality prediction models

**Fig. 1:**
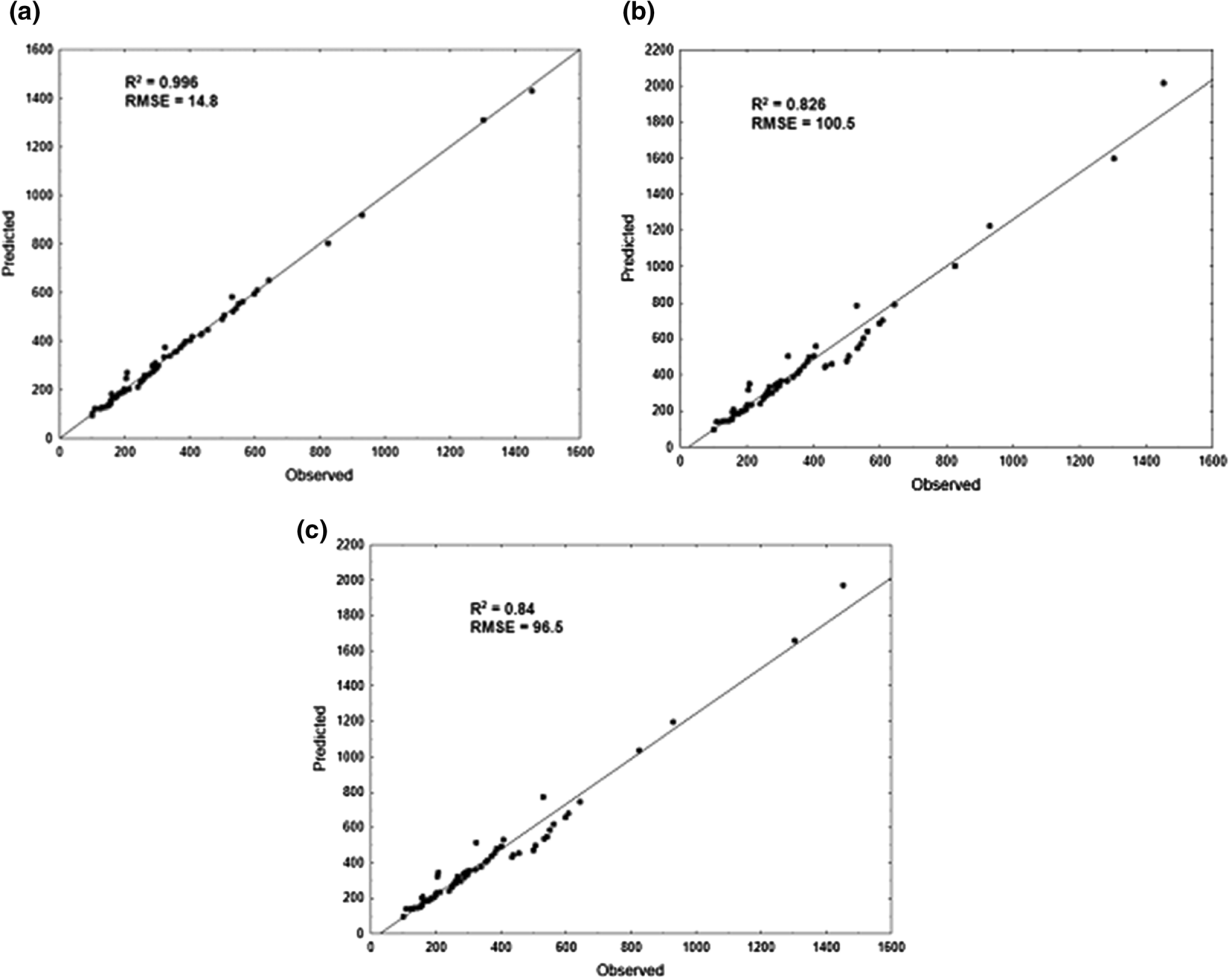
Predictability of the **a** ANN model **b** Arrhenius model **c** Log-logistic model for the TBARS value changes in meat sample enriched with plant extracts stored at 12°C. (The solid line represents a perfect match between experimental and predicted values.)

The validation of TBARS calculated through predictive model was measured by the TBARS changes of samples at 12°C. The TBARS value changes during meat samples storage predicted using these three models were plotted against the observed values (Fig. 1). The scatter plots revealed a high order of linearity which was confirmed by high adjusted regression coefficients (0.82–0.99) and low RSME values. The best prediction ability was noted for ANN model (*R*^2^ = 0.996, RMSE = 14.8). It was the model combined of all 5 best networks. The worst forecasting ability with the highest RMSE values was the Arrhenius model but the pattern was very similar to log-logistic model. It occurred in the TBARS values range from 400 to 600% of TBARS incensement. According to the R^2^ and RMSE of both models, only a slight difference in overall performance can be found between the Arrhenius model and the log-logistic one. Peleg et al. (2002) pointed out that the log-logistic model and Arrhenius model can describe the same data with similar fit. Similar observations were made by Bao et al. (2013) who modelled quality changes in Songpu mirror carp (*Cyprinus carpio*) fillets stored at chilled temperatures.

### Regression modelling (MLR)

To assess the influence of time, temperature, and addition of plant extracts addition on TBARS value increase in meat lipids fraction, multiple linear regression (MLR) was performed. The results of regression analysis are shown in Table 5. The multiple regression analysis was statistically significant with p-value equal 1.78E-18. As can be observed (Table 5), both meat storage time and temperature significantly (*p* < 0.05) affect the oxidation of intermuscular fat lipids, which is consistent with current knowledge of this process (Amaral et al. 2018; Domínguez et al. 2019). According to the regression coefficients values the best ability to inhibit oxidation process in meat samples possessed clove extract, with the highest slope value (-5164). This is supported by the research of other authors. Abdel-Aziz and Morsy (2015) successfully used clove essential oil to inhibit oxidative changes in beef burgers during frozen storage. Ground cloves were the most effective in controlling lipid oxidation, with TBARS values of 0.75 (mg/kg), after 15-d refrigerated storage of cooked ground beef (Vasavada et al. 2006). Cloves extract exhibited also antioxidant effect in raw minced pork during refrigerated storage (Muzolf-Panek et al. 2019). Also, cardamom and caraway extract showed a significant antioxidant effect in the raw pork meat system, although the extracts of these spices alone did not show strong antioxidant properties (Table 1). All plant extracts except onion extract inhibited oxidation changes in pork meat. Based on the result of this analysis it can be concluded that spices obtained from the seeds of plants showed stronger antioxidant effects than those obtained from other plant parts of different botanical origin.

**Table 5:** The results of multiple regression analysis

Independent variables and intercept	Regression coefficients	p-value
Clove	−0.5164	2.60E-35
Cardamon	−0.5030	1.07E-33
Caraway	−0.4874	7.59E-32
Thyme	−0.4651	2.73E-29
Black seed	−0.4421	9.42E-27
Allspice	−0.4197	2.22E-24
Nutmeg	−0.4052	6.76E-23
Oregano	−0.3863	4.91E-21
Basil	−0.3678	2.83E-19
Rosemary	−0.3492	1.39E-17
Bay Leaf	−0.3378	1.41E-16
Garlic	−0.3115	2.22E-14
Onion	0.0496	2.18E−01
Temperature	0.0442	9.21E-141
Time	0.1262	0.00E + 00
Intercept	4.4326	0.00E + 00

## Conclusion

This study explores the effect of temperature and antioxidant properties of selected culinary species on the secondary lipid oxidation products incensement, measured by TBARS index in raw minced pork meat stored under different temperatures. The experimental data of TBARS values were fitted to kinetic models and ANN models. The changes in TBARS were dependent on temperature well described by the first-order kinetic model. The kinetic rate constant can be modelled using Arrhenius and log-logistic models with satisfactory accuracy. To conclude, the models employed can be used for the prediction oxidative changes in the intramuscular fat fraction. The log-logistic model showed the better fit than the model based on Arrhenius equation. The best fit was noted for the model built using ANN. Additionally based on obtained parameters the antioxidant capacity of plant extracts was compered. This study demonstrated the potential usefulness of the models to realistic prediction of the TBARS changes in raw pork meat during storage. Such predictive models allow to predict oxidative changes in minced meat under different time and temperature conditions. This knowledge is very useful in designing food products and predicting the shelf-life of the products. Additionally, the effectiveness of various spices in the raw pork meat system was compared. The meat is a very complex system and, according to the research, there is no direct correlation between the antioxidant activity of the spice itself and its antioxidant effectiveness in the product.

## Data Availability

The data that support the findings of this study are available (privately) in Mendeley dataset.

## Abbreviation

TBARS: Thiobarbituric acid reactive substances

## Code availability

https://data.mendeley.com/datasets/69jfdsv8w4/draft?a=a2e5d3b7-40eb-4798-90df-7203ceb58117.
